# Nine rules to follow in collaborative research

**DOI:** 10.1192/j.eurpsy.2025.266

**Published:** 2025-08-26

**Authors:** N. Sartorius

**Affiliations:** Association for the Improvement of Mental Health Programs, Geneva, Switzerland

## Abstract

**Abstract: Introduction:**

After the exploration of factors relevant to the conduct of collaborative research it is possible to formulate a set of rules which vastly increase the probability of success in the study conducted collaboratively.

**Methods:**

A systematic exploration of the manners in which collaborative research has been organized and of the relation between the method or organization and the successful completion of the study.

**Objectives:**

The goal of this exploration was to formulate a set of rules which should be followed in conducting multicenter collaborative research to enhance the probability of its success.

**Results:**

The examination of the experience in the conduct of the studies organized and lead by the author it was possible to formulate certain rules which, when followed, not only led to a successful completion of the studies but also led to a number of additional benefits for the centres in which the study was conducted. The nine principal rules which were formulated in this way will be presented.

**Conclusions:**

Collaborative research is more likely to have useful results if it is developed bearing certain rules in mind.

**Disclosure of Interest:**

None Declared

